# Dietary Patterns and Nutritional Factors in Multiple Sclerosis: A Narrative Review

**DOI:** 10.7759/cureus.114634

**Published:** 2026-08-17

**Authors:** Ahmad Mufti, Reem Z Almarhabi, Wejdan Bajandouh, Rahma M Alhasani, Gufran Kambiji, Teyf M Althubiani, Ruba J Almahmudi, Ftoon Alshaddadi, Wesam A Nasif, Abeer S Ali

**Affiliations:** 1 Medical Genetics, Umm al-Qura University, Makkah, SAU; 2 College of Medicine, Umm al-Qura University, Makkah, SAU; 3 Medicine, Umm al-Qura University, Makkah, SAU; 4 Biochemistry, Umm al-Qura University, Makkah, SAU; 5 Molecular Biology, Faculty of Biotechnology, Sadat City University, Sadat, EGY; 6 Pathology, Makkah National College, Makkah, SAU

**Keywords:** and wahls, "clinical outcomes", food and dietary patterns, inflammatory mediators, mediterranean diet (md), s: multiple sclerosis, swank

## Abstract

Multiple sclerosis (MS) is a chronic immune-mediated disorder of the central nervous system characterized by inflammation, demyelination, and progressive neurological dysfunction. Increasing attention has been given to the potential role of diet as an adjunctive strategy for improving symptoms, modulating inflammation, and enhancing quality of life in people with MS. This narrative review examines current evidence from clinical trials, observational studies, and relevant reviews on the effects of dietary patterns and selected nutritional components on clinical outcomes in MS, with particular focus on the Mediterranean diet, the Swank diet, the Wahls diet, ketogenic diets, plant-based diets, and individual components such as omega‑3 fatty acids, antioxidants, and saturated fats. Available data suggest that some dietary interventions may be associated with improvements in fatigue, quality of life, metabolic parameters, and, in certain studies, disability-related outcomes. Overall, patterns rich in fruits, vegetables, whole grains, unsaturated fats, and anti-inflammatory nutrients, and low in saturated fat, appear most consistently beneficial, although findings remain heterogeneous and limited by methodological variability. Proposed mechanisms include modulation of systemic inflammation, reduction of oxidative stress, favorable alterations in gut microbiota composition, and support of metabolic health. Diet may therefore represent a useful adjunct to conventional MS management, particularly when centered on balanced, anti-inflammatory Mediterranean-style or plant-rich patterns, but it should not be regarded as a substitute for established pharmacological therapies. Larger, long-term, and methodologically rigorous randomized trials are still needed to clarify the efficacy, safety, and sustainability of specific dietary interventions and to define clear, evidence-based dietary recommendations for people living with MS.

## Introduction and background

Multiple sclerosis (MS) is a chronic, immune-mediated disease of the central nervous system characterized by inflammatory demyelination, axonal injury, and progressive neurological impairment. It commonly affects young and middle-aged adults and may present with a wide range of symptoms, including fatigue, sensory disturbance, gait impairment, muscle weakness, visual symptoms, and cognitive dysfunction, all of which may substantially reduce quality of life. Although the precise aetiology of MS remains incompletely understood, it is widely accepted that the disease arises from a complex interaction between genetic susceptibility and environmental influences [1].

The pathogenesis of MS is characterized by dysregulated immune activation involving both the innate and adaptive immune systems. In genetically susceptible individuals, environmental factors such as viral infections, vitamin D deficiency, smoking, and obesity may trigger the activation of autoreactive CD4⁺ and CD8⁺ T lymphocytes, which migrate across the blood-brain barrier and initiate inflammatory cascades that lead to demyelination, axonal damage, and neurodegeneration. B lymphocytes further contribute by presenting antigens, secreting pro-inflammatory cytokines, and producing intrathecal immunoglobulins. Although disease-specific circulating autoantibodies have not been established as reliable diagnostic biomarkers in MS, the presence of cerebrospinal fluid oligoclonal IgG bands remains an important indicator of intrathecal immune activation and supports the diagnosis. Given the central role of chronic inflammation and immune dysregulation in MS, interventions capable of modulating immune responses, including dietary factors, have attracted increasing research interest as potential adjuncts to disease-modifying therapies [1-3].

In addition to pharmacological treatment, there is growing interest in modifiable lifestyle factors that may influence disease burden in MS, particularly diet. Nutritional factors have been investigated for their potential effects on inflammatory pathways, oxidative stress, immune regulation, gut microbiota composition, and metabolic status, all of which may be relevant to the pathophysiology and clinical expression of MS. As a result, dietary strategies have increasingly been explored as supportive measures that may complement standard disease-modifying therapies rather than replace them [1,2].

A number of dietary approaches have attracted attention in the MS literature, including the Mediterranean diet, the Swank diet, the Wahls diet, ketogenic diets, and whole-food plant-based dietary patterns. In parallel, interest has also grown in the role of individual nutritional components such as omega-3 fatty acids, antioxidants, dairy products, and gluten-related factors. However, the evidence remains variable in quality, and findings are often difficult to compare because of differences in study design, intervention duration, outcome measures, and patient populations [3].

Despite growing interest in dietary interventions for MS, the available evidence remains heterogeneous, with considerable variation in dietary approaches, study designs, outcome measures, and methodological quality. In addition, evidence relating to dietary patterns, individual nutritional components, and their potential biological mechanisms is often presented separately, making it difficult to obtain a comprehensive and clinically relevant overview. This narrative review therefore aims to examine the current evidence on the relationship between diet and MS, with particular emphasis on dietary patterns, selected nutritional components, and possible biological mechanisms, and their implications for patient care. The review aimed to summarize findings from the literature concerning the effects of diet on MS-related outcomes, including fatigue, quality of life, disability, inflammatory activity, and metabolic health. It also sought to identify areas of uncertainty, highlight the limitations of the existing literature, and provide a balanced synthesis to help inform clinical practice and future research.

Methods

Electronic searches were conducted in PubMed/MEDLINE (MEDical Literature Analysis and Retrieval System Online), Google Scholar, and other relevant sources such as reference lists of key articles using combinations of keywords including “multiple sclerosis”, “diet”, “dietary intervention”, “Mediterranean diet”, “Swank”, “Wahls”, “ketogenic”, “plant-based”, “gluten-free”, “omega-3”, “antioxidants”, “flavonoids”, “gut microbiota”, and “oxidative stress”. The review focused particularly on dietary approaches frequently discussed in the MS literature, including the Mediterranean diet, the Swank diet, the Wahls diet, ketogenic diets, plant-based diets, and gluten-free diets, together with specific nutritional components such as omega-3 fatty acids, flavonoids, dairy products, and saturated fats. In addition to patient-centered outcomes, evidence relating to putative mechanisms such as modulation of inflammation, oxidative stress, gut microbiota, lipid metabolism, and myelin-related processes was considered where appropriate.

The literature search primarily included articles published between 2019 and February 2026. Earlier landmark publications were also considered when necessary to provide historical background or foundational evidence. Relevant literature included clinical trials, observational studies, systematic reviews, and other review articles addressing dietary patterns or nutritional components in people with MS. Experimental and mechanistic studies were also included when they provided relevant insights into the biological effects of nutrition on MS pathophysiology. Publications that were not related to MS, did not address dietary or nutritional factors, or were not considered relevant to the objectives of this review, and conference abstracts without sufficient methodological detail were not included.

As this was a narrative review, study identification and selection were performed by the authors through iterative evaluation of the retrieved literature based on relevance and scientific contribution rather than through a predefined systematic review protocol. The selected literature was interpreted descriptively and organized thematically to provide an accessible overview of current evidence and ongoing areas of uncertainty in this field. The thematic organization adopted throughout the review was developed iteratively based on the dietary patterns most frequently investigated in the literature and their commonly reported clinical outcomes, rather than according to a predefined analytical framework.

## Review

Dietary patterns and interventions in MS

Dietary patterns have attracted considerable attention in MS because they represent modifiable lifestyle factors that may influence symptom burden, inflammatory activity, and overall health status. A wide range of dietary approaches has been proposed in the MS literature, ranging from broadly health-promoting patterns such as Mediterranean and plant-based diets to more restrictive regimens such as the Swank, Wahls, ketogenic, and gluten-free diets. Although these approaches differ in their composition and theoretical rationale, most aim to reduce inflammation, improve metabolic health, and alleviate common MS-related symptoms, particularly fatigue and reduced quality of life. The following sections summarize the principal dietary patterns investigated in MS and evaluate the strength and limitations of the available evidence for each.

Mediterranean and Mediterranean-like Diets

The Mediterranean diet is characterized by a high intake of fruits, vegetables, whole grains, legumes, nuts, and olive oil, moderate consumption of fish and other sources of unsaturated fats, and limited intake of red meat, processed foods, and saturated fat. This dietary pattern has received considerable attention in MS because of its recognized anti-inflammatory, antioxidant, and cardiometabolic benefits, which may be relevant to disease activity and overall patient wellbeing [4-6].

Observational studies and recent reviews suggest that greater adherence to Mediterranean-like dietary patterns may be associated with lower fatigue severity, better quality of life, less objective disability, and improved cognitive outcomes in people with MS. A systematic review published in 2024 found possible beneficial effects on inflammatory status, fatigue, quality of life, attack rate, and cognitive dysfunction, although it also noted that the number of well-designed randomized controlled trials remains limited [7]. These findings support the view that Mediterranean-style eating may be one of the most clinically relevant dietary approaches in MS, while also showing that stronger intervention studies are still needed.

Several biological mechanisms may explain these associations. Mediterranean-style diets are rich in monounsaturated and polyunsaturated fats, fiber, vitamins, and polyphenols, all of which may contribute to reduced systemic inflammation, lower oxidative stress, healthier gut microbiota composition, and improved metabolic regulation. Together, these effects may help create a physiological environment that is more favorable for symptom control and long-term neurological health in individuals with MS [2,3,8,9].

Swank and Wahls Diets

The Swank diet and the Wahls diet are among the most frequently discussed disease-specific dietary approaches in MS. The Swank diet is a low-saturated-fat dietary pattern that emphasizes fruits, vegetables, grains, and limited intake of saturated fats, whereas the Wahls diet is a modified Paleolithic elimination diet that encourages high consumption of vegetables and excludes foods such as gluten-containing grains, dairy, and, in some versions, legumes [10]. Because both diets are widely recognized within the MS community, they are often discussed as targeted nutritional strategies for symptom management rather than as general healthy eating patterns [11,12].

The strongest comparative evidence comes from the WAVES randomized parallel-arm clinical trial, which evaluated the effects of the Swank and Wahls diets in individuals with relapsing-remitting MS. In this trial, both dietary interventions were associated with clinically meaningful reductions in fatigue and improvements in quality of life at 12 weeks, with benefits maintained or further improved at 24 weeks [13]. The Wahls diet showed larger gains in some quality-of-life measures, particularly mental quality-of-life scores, while both diets produced favorable changes in fatigue severity. Secondary analyses also found reductions in weight, body mass index, total cholesterol, and low-density lipoprotein cholesterol in both groups, although the reduction in fatigue did not appear to be directly explained by improvements in metabolic markers alone [14].

Despite these encouraging findings, the evidence base for both diets, the Swank and Wahls diets, remains limited and should be interpreted cautiously. Much of the published interventional evidence comes from the same research programme, sample sizes have been modest, and long-term adherence may be challenging because both diets involve varying degrees of restriction. In addition, concerns have been raised regarding nutritional adequacy, particularly when restrictive versions of these diets are followed without professional supervision, as some participants may be at risk of inadequate intake of selected micronutrients. Overall, the Swank and Wahls diets appear promising for fatigue and quality-of-life outcomes in MS, but stronger independent studies with longer follow-up are still required before either diet can be recommended with confidence as a disease-specific standard of care [14,15].

Plant-Based Diets

Plant-based diets have also received increasing attention as a potential supportive strategy in MS, particularly because they are typically rich in fiber, antioxidants, vitamins, and unsaturated fats, while being lower in saturated fat and highly processed foods. These characteristics make them biologically plausible candidates for reducing inflammation, improving metabolic health, and influencing gut microbiota composition, all of which may be relevant to MS pathophysiology and symptom burden [16].

Clinical evidence suggests that low-fat plant-based dietary interventions may improve selected patient-reported outcomes, especially fatigue, body weight, and metabolic markers, although effects on core disease activity remain less clear [16,17]. In a randomized controlled trial, a very-low-fat plant-based diet was found to be safe and well tolerated, and was associated with improvements in fatigue, body mass index, and lipid profiles over 12 months, but it did not produce significant changes in MRI outcomes, relapse rate, or disability scores during the study period [18]. Reviews of dietary interventions in MS have similarly concluded that plant-based diets appear promising, particularly for fatigue and general health, but that current evidence is insufficient to confirm a disease-modifying effect [16-18].

The potential benefits of plant-based diets may arise through several complementary mechanisms. High intake of fruits, vegetables, legumes, and whole grains may enhance antioxidant capacity, reduce oxidative stress, improve lipid metabolism, and promote a more favourable intestinal microbial profile, which in turn could influence immune regulation and inflammatory activity [16,17]. However, the evidence base remains limited by small sample sizes, heterogeneity of dietary protocols, and the relative scarcity of long-term controlled trials. Consequently, plant-based diets may be best regarded at present as a potentially beneficial adjunct to standard MS care rather than a replacement for evidence-based pharmacological treatment [16-19].

Ketogenic and Low-Carbohydrate Diets

Ketogenic diets are very low-carbohydrate, high-fat dietary patterns designed to induce nutritional ketosis, whereas broader low-carbohydrate diets restrict carbohydrate intake to a lesser degree while increasing fat and/or protein intake [11,20]. These approaches have attracted interest in MS because of their potential to modulate energy metabolism, reduce adiposity, influence inflammation, and alter gut microbiota composition. However, they are more restrictive than Mediterranean or general plant-based diets and therefore require closer clinical supervision [21-23].

Emerging clinical evidence suggests that ketogenic diets may improve several MS-related symptoms over the short term. In a prospective phase II study of relapsing MS, a six‑month ketogenic intervention was reported to be safe and tolerable, with high adherence and significant reductions in body fat, fatigue, and depression, alongside improvements in quality-of-life scores and neurological disability measures [21]. Other pilot work and patient-reported data similarly indicate perceived benefits in fatigue, mood, and functional capacity in some individuals, although most studies to date have been small, of limited duration, and without long-term follow-up. Reviews emphasize that while short-term results are promising, robust randomized controlled trials comparing ketogenic diets with more conventional dietary patterns in MS are still lacking [23].

Proposed mechanisms by which ketogenic and low-carbohydrate diets might influence MS outcomes include reductions in systemic inflammation, improved mitochondrial function, changes in adipokine profiles, and favorable alterations in the gut microbiome, alongside weight loss and better metabolic control. Nevertheless, these diets may also carry potential risks, such as nutrient deficiencies, gastrointestinal side effects, or challenges in long-term adherence, particularly in individuals with comorbid conditions. Current guidance from MS organizations therefore tends to view ketogenic and strict low-carbohydrate diets as experimental options that should only be undertaken with medical and dietetic supervision, rather than as routine recommendations for all patients with MS [24,25].

**Figure 1:**
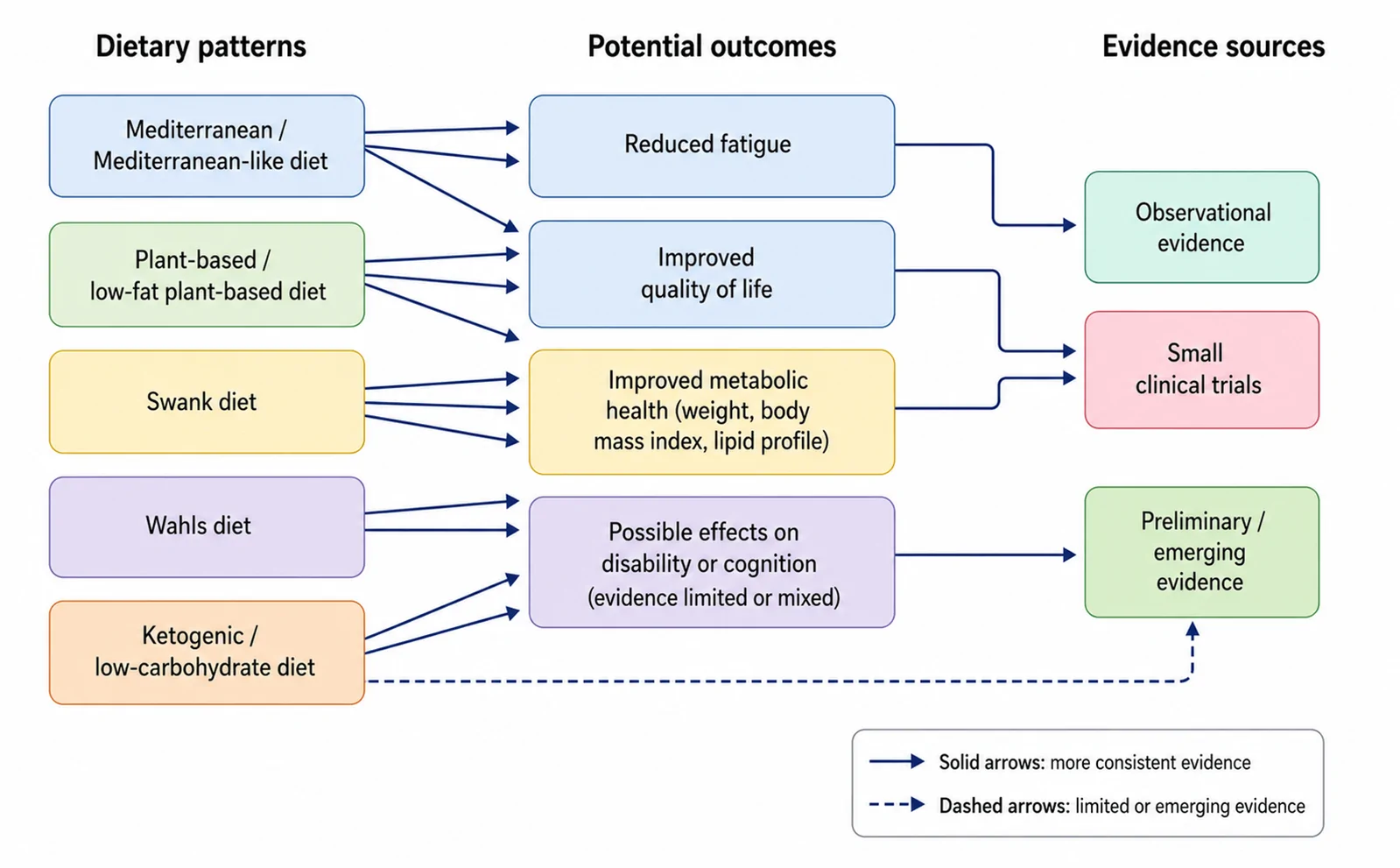
Overview of dietary patterns, reported clinical outcomes, and the strength of available evidence in multiple sclerosis (MS). Solid arrows indicate associations supported by relatively more consistent findings, whereas dashed arrows indicate limited or emerging evidence. The figure is intended as a conceptual summary of the literature reviewed and does not imply causal relationships. Image Credit: Authors

Figure 1 summarizes the principal dietary patterns discussed in this review and their reported associations with fatigue, quality of life, metabolic health, disability, and cognitive function.

Specific nutrients and food components

Omega-3 Fatty Acids and Fish Oils

Omega-3 polyunsaturated fatty acids (n‑3 PUFAs), particularly eicosapentaenoic acid (EPA) and docosahexaenoic acid (DHA), have long been investigated in MS because of their anti‑inflammatory and neuroprotective properties. They are typically derived from oily fish or fish-oil supplements and have been proposed to modulate immune responses, reduce production of pro-inflammatory cytokines, and support myelin integrity [16].

Clinical studies of omega-3 supplementation in MS have produced mixed results. Some trials and observational data suggest modest benefits on relapse rate, disability progression, or fatigue, whereas others have not demonstrated significant effects when omega‑3s are added to modern disease-modifying therapies [26]. Overall, reviews conclude that omega-3 supplementation appears safe and may confer small or adjunctive benefits, but the evidence is insufficient to recommend it as a primary disease-modifying strategy in MS, and any use should be considered within the broader context of cardiovascular risk reduction and general health [16,17].

Antioxidants and Flavonoids

Antioxidant nutrients and bioactive compounds, including flavonoids found in fruits, vegetables, tea, and other plant foods, have also been explored for their potential role in MS. Preclinical studies and experimental models suggest that flavonoids such as luteolin, quercetin, and related molecules may reduce oxidative stress, inhibit microglial activation, and downregulate pro-inflammatory signaling pathways, thereby attenuating neuroinflammation and demyelination [27].

Human evidence remains limited but indicates that higher intakes of flavonoid-rich foods and antioxidant-containing diets may be associated with better symptom control and improved quality of life in people with MS, although these findings are often embedded within broader dietary interventions rather than isolated flavonoid supplementation. Current reviews emphasize that encouraging a diet rich in fruits and vegetables is reasonable for overall health and may provide additional neuroprotective benefits, but specific antioxidant or flavonoid supplements cannot yet be recommended as stand-alone therapies due to insufficient high-quality clinical trials [28].

Saturated Fats and Vegetable Oils

Saturated fat intake and the types of dietary oils consumed have been implicated in MS since the early work of Swank and others, who proposed that diets low in saturated fat might reduce relapse frequency and disability progression. Subsequent observational studies have supported associations between higher saturated fat intake, more “Western” dietary patterns, and worse MS-related outcomes, whereas diets emphasizing unsaturated fats, including certain vegetable oils and fish oils, appear more favorable [12,16,29,30].

However, more recent analyses highlight that the relationship between specific oils and MS outcomes is complex, and that not all vegetable oils are equivalent: some may contribute beneficial unsaturated fats, while others, particularly those high in saturated or trans fats, may exacerbate inflammation. Current guidance therefore focuses on reducing overall saturated fat intake and replacing it with sources of monounsaturated and polyunsaturated fats, such as olive oil, nuts, seeds, and oily fish, rather than promoting any single “MS oil” as a therapeutic intervention [31,32]. Key clinical studies of dietary interventions in multiple sclerosis are summarized in Table 1.

**Table 1:** Representative studies of dietary interventions in multiple sclerosis (MS) QoL: quality of life; RCT: randomized controlled trial; TNF-α: tumor necrosis factor-alpha; IL: interleukin; EDSS: Expanded Disability Status Scale

Study	Study design and population	Dietary intervention	Comparator	Duration	Main findings	Key limitations
Abbasi et al., 2024 [7]	Systemic review	Mediterranean-like diet	Control groups (in RCTs) or MS cohorts with lower Mediterranean diet adherence (in observational studies).	Databases searched up to March 2023; individual study durations vary	Improved inflammatory status, fatigue, QoL, attack rate, and cognition.	Limited number of well-designed RCTs; high heterogeneity in study designs, outcomes, and intervention groups preventing a meta-analysis.
Titcomb et al., 2021 [13]	Randomized parallel-arm clinical trial in people with relapsing-remitting multiple	Wahls elimination diet	Swank low-saturated-fat diet	24 weeks after run-in	Both diets reduced fatigue and improved QoL; the Wahls diet showed larger gains in some QoL outcomes	Modest sample size; short follow-up; adherence and long-term generalizability remain uncertain
Crippes et al., 2023 [14]	Secondary analysis of the WAVES randomized parallel-arm trial in relapsing-remitting MS	Swank and Wahls diets	Between-diet comparison within WAVES	Trial follow-up analysis	Diet-induced changes in functional disability appeared to be mediated by fatigue improvement	Secondary analysis from the same trial programme; limited long-term outcome data
Snetselaar et al., 2023 [17]	Systematic review and network meta-analysis of randomized trials	Dietary interventions targeting fatigue and quality of life	Randomized trial comparisons	Review article	Found comparative evidence regarding effects of dietary interventions on fatigue and QoL in MS	Limited by the number, size, and heterogeneity of available randomized trials
Yadav et al., 2016 [18]	RCT in people with MS	Low-fat, plant-based diet	Usual diet/control condition	12 months	Improved fatigue, BMI, and lipid profile; no significant effect on MRI lesions, relapse rate, or disability during the study period	Limited sample size and limited effect on core neurological endpoints
Cuthbert et al., 2022 [19]	Two-case report of people living with MS	Whole-food plant-based diet with medication cessation	No formal comparator	Case-based follow-up	Described clinical improvement after transition to a whole-food plant-based diet	Very low level of evidence; uncontrolled case report; medication changes confound interpretation
Brenton et al., 2022 [21]	Phase II study in relapsing MS	Ketogenic diet	Single arm/non-standard comparison	6 months	Reported safety, tolerability, and potential clinical benefits including improvements in fatigue, mood, adiposity and QoL	Small study with limited long-term follow-up and no definitive efficacy conclusion
Wetmore et al., 2023 [20]	Post-trial follow-up/patient-perception study in relapsing MS	Ketogenic diet	Post-trial observational assessment	Post-trial follow-up	Reported patient perceptions, adherence patterns, and selected outcomes after ketogenic dietary intervention	Observational follow-up; subject to self-report and selection bias
Ramirez-Ramirez et al., 2013 [25]	Randomized, double- blind, placebo- controlled trial	Fish oil	Placebo group	12 months	Significant reduction in serum TNF-α, IL-1\β interferon, IL-6, and nitric oxide; no effect on EDSS or relapse rates.	Small sample size for clinical outcomes; relapse severity/intervals not tracked

Mechanisms linking diet and MS

Inflammation and Immune Modulation

Chronic inflammation is central to the pathophysiology of MS, and one of the main reasons diets have attracted interest in this condition is their potential to influence immune and inflammatory pathways. Diets rich in fruits, vegetables, whole grains, unsaturated fats, and other minimally processed foods may help reduce the production of pro-inflammatory mediators, whereas dietary patterns that are high in saturated fat, refined sugars, and ultra-processed foods may promote a more pro-inflammatory internal environment. This provides a plausible biological basis for the observed associations between healthier dietary patterns and improved fatigue, quality of life, and general wellbeing in people with MS [11,32,33].

Oxidative Stress

Oxidative stress is another important mechanism implicated in MS, arising from an imbalance between reactive oxygen species and the body’s antioxidant defenses. Excess oxidative stress may contribute to demyelination, axonal injury, mitochondrial dysfunction, and progression of neurological disability in MS. Diets rich in antioxidant-containing foods, including vegetables, fruits, legumes, nuts, and polyphenol-rich plant foods, may therefore help limit oxidative damage and support cellular resilience. This is one reason why Mediterranean-style and plant-rich dietary patterns are often considered biologically favorable in MS, even when direct clinical evidence remains incomplete [30].

Gut Microbiota and the Gut-Brain Axis

Growing evidence suggests that the gut microbiota plays an important role in immune regulation and may influence MS through the gut-brain axis. People with MS have been shown to exhibit alterations in gut microbial composition compared with healthy individuals, and these changes may contribute to inflammatory signaling, impaired intestinal barrier function, and altered production of metabolites such as short-chain fatty acids. Because diet is one of the strongest modulators of the intestinal microbiome, dietary interventions rich in fiber and plant-derived nutrients may help promote a more favorable microbial environment and, in turn, influence immune homeostasis. Although this area remains mechanistically complex, it provides a strong conceptual link between dietary patterns and MS-related outcomes [33-37].

Myelin Integrity and Lipid Biology

MS is characterized by immune-mediated damage to myelin, the lipid-rich sheath that surrounds nerve fibers and supports efficient nerve conduction. Nutritional factors may influence this process indirectly through effects on inflammation and oxidative stress, and possibly more directly through lipid metabolism and the biological environment required for remyelination. Emerging mechanistic work suggests that disturbances in fatty-acid metabolism and lipid signaling may contribute to neuroinflammation following myelin injury, further supporting interest in dietary fat quality rather than simple fat quantity alone. This helps explain why diets low in saturated fat and richer in unsaturated fats are often emphasized in the MS nutrition literature [32,38-40].

Energy Metabolism and Metabolic Health

Metabolic dysfunction may also influence symptom burden and disease expression in MS, particularly through pathways involving obesity, dyslipidemia, insulin resistance, and mitochondrial impairment. Dietary patterns that improve body weight, lipid profiles, and overall metabolic health may therefore provide indirect benefits, even if they do not directly alter core disease activity. This may partly explain why some dietary interventions improve fatigue and quality-of-life measures even when their effects on MRI findings, relapse rates, or disability progression are less consistent. In this context, diet may be viewed not only as a possible immunological modifier, but also as a means of improving the broader metabolic environment in which MS develops and progresses [7,21,33,34].

**Figure 2:**
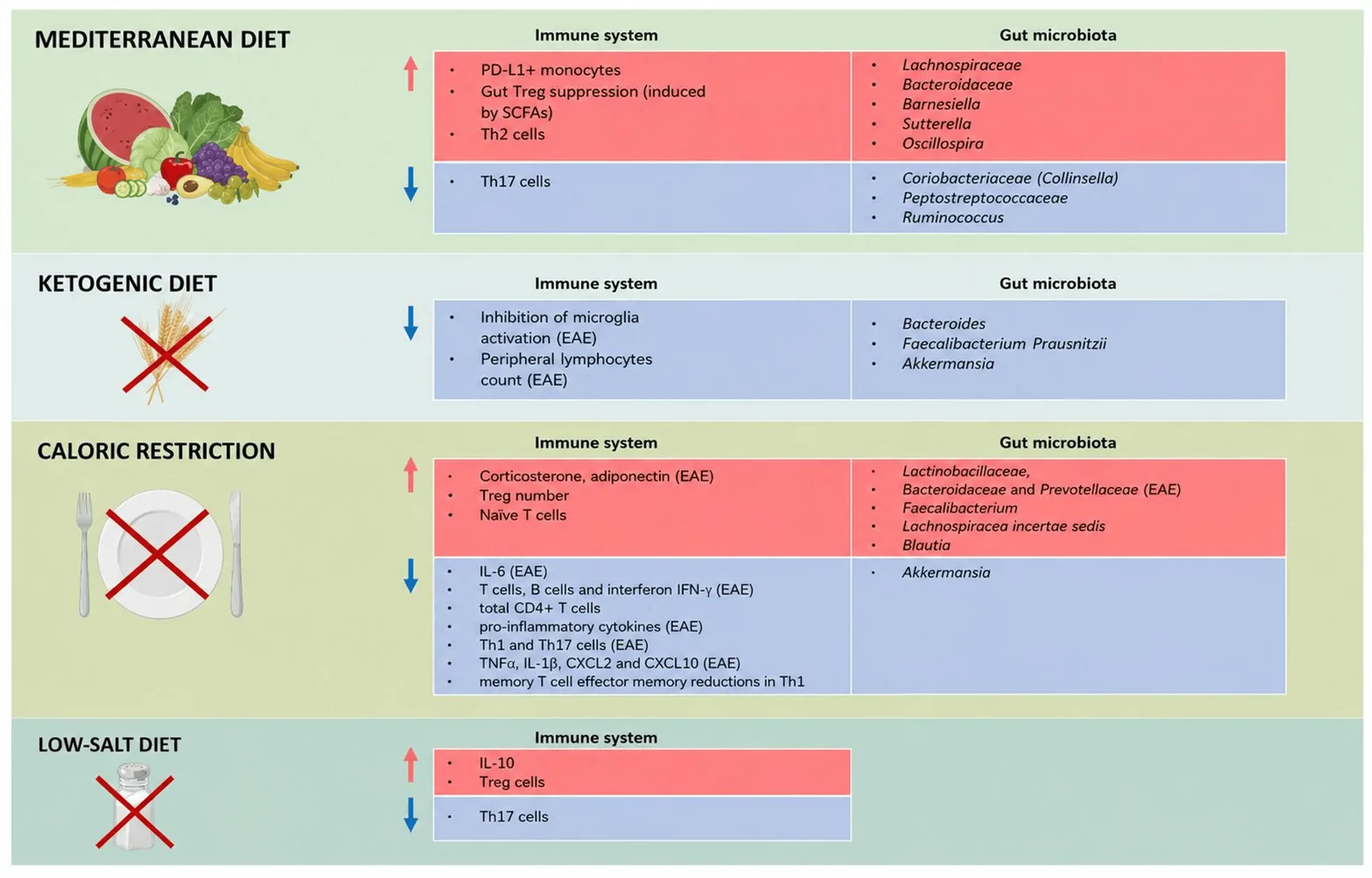
Impact of dietary interventions on immune responses and gut microbiota in multiple sclerosis (MS). Effects of the Mediterranean diet (MD; first panel), the ketogenic diet (KD; second panel), calorie restriction (CR; third panel), and low-salt diet (fourth panel) on the immune system and the gut microbiota in MS. Different diets affect the immune system and the gut microbiota by increasing (red) and decreasing (blue) the number of cells, cytokines, and microorganisms. Image Source: Bronzini et al. [40]; used under the terms of the CC BY 4.0 Attribution 4.0 International Deed (https://creativecommons.org/licenses/by/4.0/)

The proposed pathways through which dietary patterns and specific nutrients may influence MS are summarized in Figure 2 [40].

Clinical implications

Dietary patterns appear to have an important supportive role in the overall management of MS, particularly in relation to fatigue, quality of life, and general cardiometabolic health. Based on current evidence, Mediterranean-style and other plant-rich dietary patterns, which emphasize fruits, vegetables, whole grains, legumes, nuts, and unsaturated fats while limiting saturated fat and ultra-processed foods, are the most consistently associated with favorable outcomes in people with MS. Such patterns are also compatible with established recommendations for cardiovascular and metabolic risk reduction, which is particularly relevant because many individuals with MS have comorbidities that can influence long-term disability and mortality [11,16,41-43].

More specialized diets such as the Swank and Wahls diets, as well as ketogenic and low-carbohydrate approaches, may provide additional benefits for some patients, particularly in terms of fatigue and quality of life, but they are more restrictive and have a less robust evidence base. These diets are best considered adjunctive options that should only be undertaken with appropriate medical and dietetic supervision, especially in patients with existing nutritional risks or complex comorbidities. Clinicians should encourage people with MS to avoid delaying or replacing disease-modifying therapies in favor of unproven dietary interventions, and instead frame diet as one component of a comprehensive management plan that also includes pharmacological treatment, physical activity, and attention to mental health [21,29,43].

Limitations of the evidence and future directions

Despite growing interest and a rapidly expanding literature, the evidence base on diet in MS remains limited by several important factors. Many clinical trials of dietary interventions have small sample sizes, short follow-up periods, and heterogeneous designs, which restricts the ability to draw firm conclusions about long-term effects on relapse rate, MRI outcomes, or disability progression. Adherence to restrictive dietary regimens is often difficult to sustain, and many studies rely on self-reported dietary intake, which may introduce bias and reduce the accuracy of exposure assessment. In addition, a substantial proportion of evidence for certain diets, such as the Swank and Wahls diets, comes from single research groups, raising questions about generalizability and the need for independent replication 

Future research should therefore prioritize adequately powered, long-term randomized controlled trials comparing well-defined dietary interventions with appropriate control conditions in diverse MS populations. Studies that combine rigorous clinical outcome measures with mechanistic endpoints, such as inflammatory markers, oxidative stress indices, gut microbiota profiles, and neuroimaging findings, are particularly important for clarifying how diet might influence MS pathophysiology. There is also a need for pragmatic trials and implementation studies that explore the feasibility, safety, and sustainability of dietary changes in real-world settings, as well as mixed-methods research to understand patient perspectives and information needs. Together, these efforts will help to move the field from promising but preliminary observations towards evidence-based, practical dietary guidance for people living with MS. 

## Conclusions

Diet has become an area of increasing research interest in the supportive management of MS, with emerging evidence suggesting that certain dietary patterns may be associated with improvements in fatigue, quality of life, inflammatory status, and broader metabolic health. Among the dietary approaches examined, Mediterranean-style and plant-rich diets have shown the most consistent associations with favorable outcomes, whereas more restrictive interventions, including the Swank, Wahls, and ketogenic diets, remain promising but are supported by more limited and less generalizable evidence.

At present, no specific diet can be recommended as a disease-modifying treatment for MS, and dietary interventions should not be viewed as a substitute for established disease-modifying therapies. Rather, healthy dietary modification may serve as a supportive component of comprehensive MS care by helping to improve symptom burden, promote cardiometabolic health, and enhance overall wellbeing. Overall, the current evidence supports considering diet as an adjunctive strategy within a multidisciplinary approach to MS management. However, given the heterogeneity and methodological limitations of the available literature, further large-scale, long-term, and well-designed studies are required to determine the efficacy, safety, and sustainability of specific dietary interventions and to establish evidence-based nutritional recommendations for people with MS.
